# Supplementary material for: Phytochemical Analysis, Antioxidant and Enzyme-Inhibitory Activities, and Multivariate Analysis of Insect Gall Extracts of Picea koraiensis Nakai
Source: Molecules. 2023 Aug 11;28(16):6021. doi: 10.3390/molecules28166021 (PMC10459859; doi:10.3390/molecules28166021)
Supplement: Supplementary file 1 [file molecules-28-06021-s001.zip › molecules-2511007-supplementary.pdf]

SUPPLEMENTARY MATERIAL

Phytochemical analysis, antioxidant and enzyme-inhibitory activities and multivariate analysis of insect gall extracts of *Picea koraiensis* Nakai

Yanqiu Wang [1,2,†], Hui Sun [3,†], Xu He [1,3], Meihua Chen [1,3], Hao Zang [1,3], Xuekun Liu [1,3,*] and Huri Piao [1,*]

[1] College of Pharmacy, Yanbian University, Yanji 133000, China
[2] Tonghua Health School, Tonghua 134000, China
[3] Green Medicinal Chemistry Laboratory, School of Pharmacy and Medicine, Tonghua Normal University, Tonghua 134002, China
* Correspondence: liuxuekunth@126.com (X.L.); piaohr@ybu.edu.cn (H.P.);
Tel.: +86-435-320-2678 (X.L.); +86-433-243-5003 (H.P.)
† These authors contributed equally to this work.

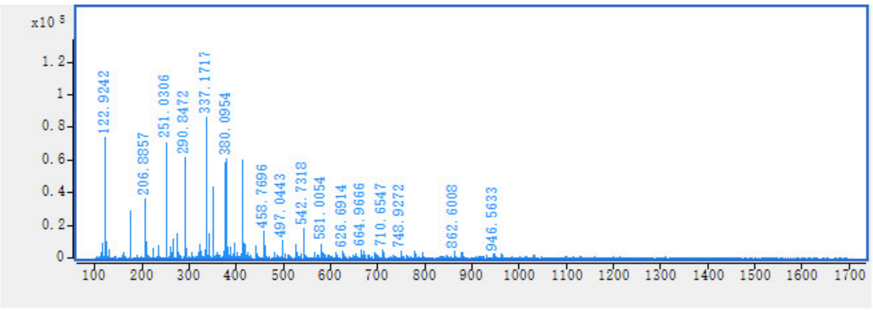

Figure S1 MS spectrum of peak 1

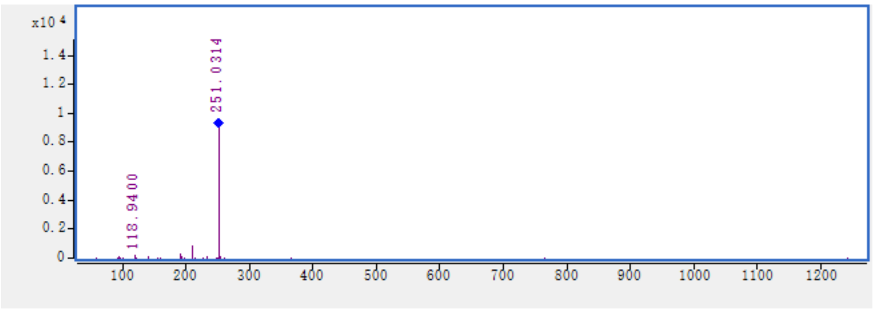

Figure S2 MS/MS spectrum of peak 1

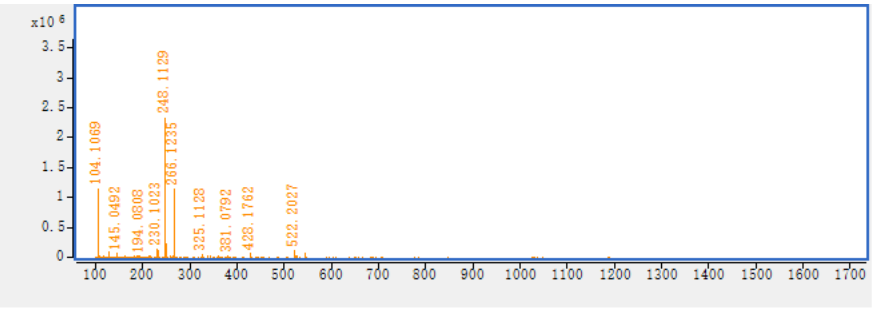

Figure S3 MS spectrum of peak 2

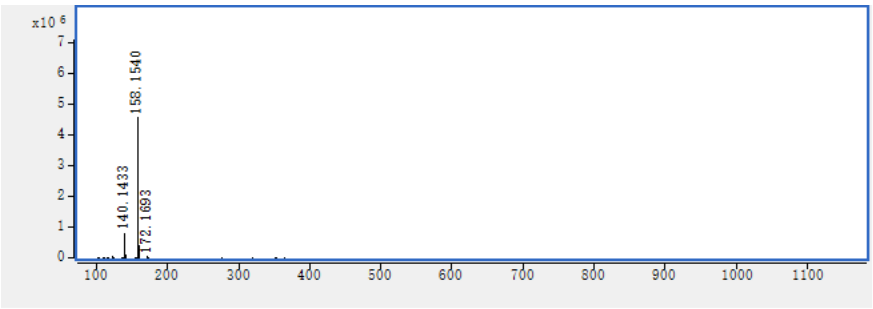

Figure S4 MS spectrum of peak 3

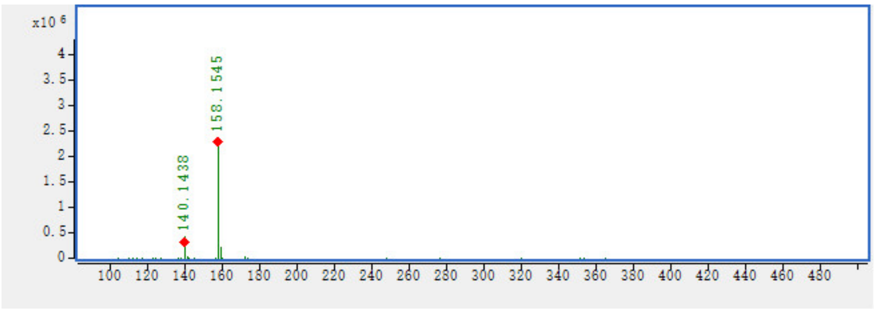

Figure S5 MS/MS spectrum of peak 3

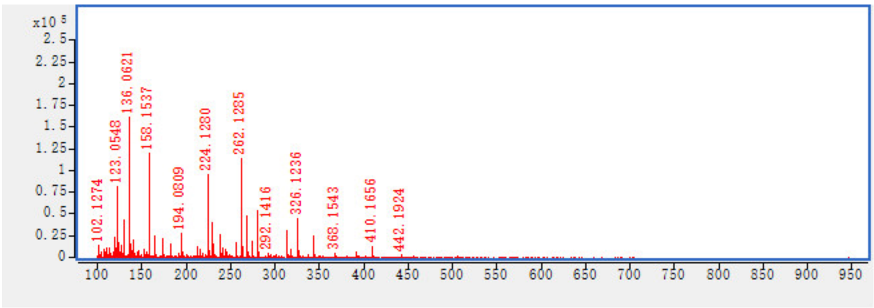

Figure S6 MS spectrum of peak 4

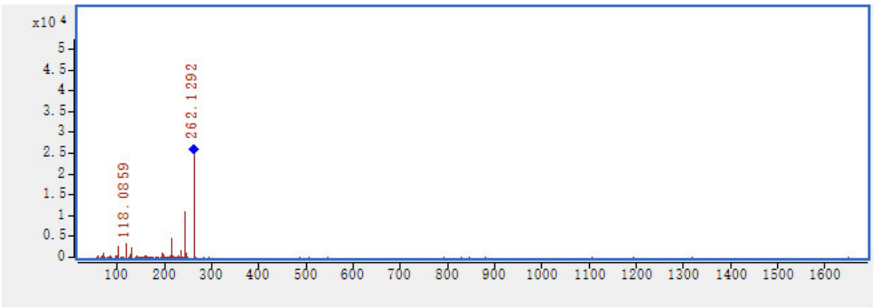

Figure S7 MS/MS spectrum of peak 4

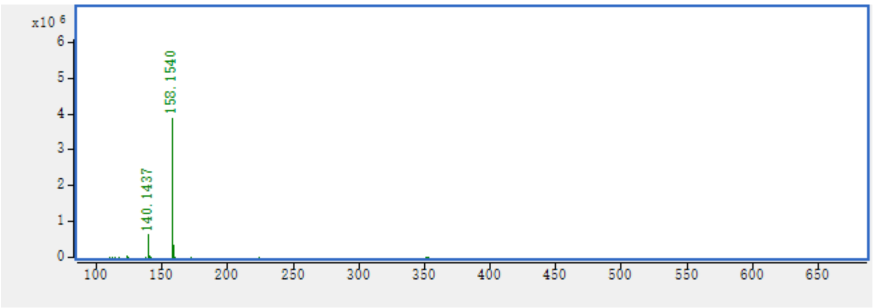

Figure S8 MS spectrum of peak 5

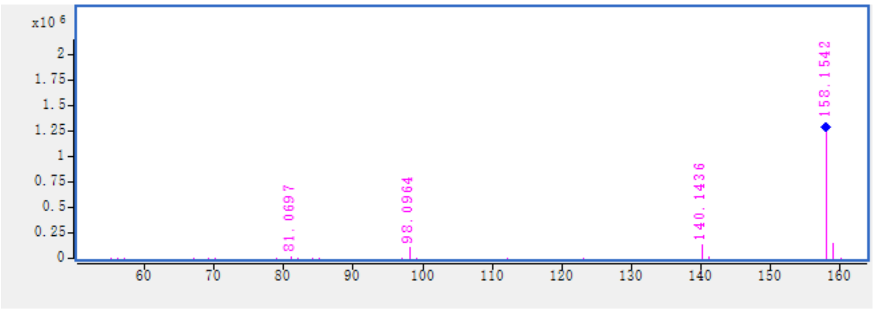

Figure S9 MS/MS spectrum of peak 5

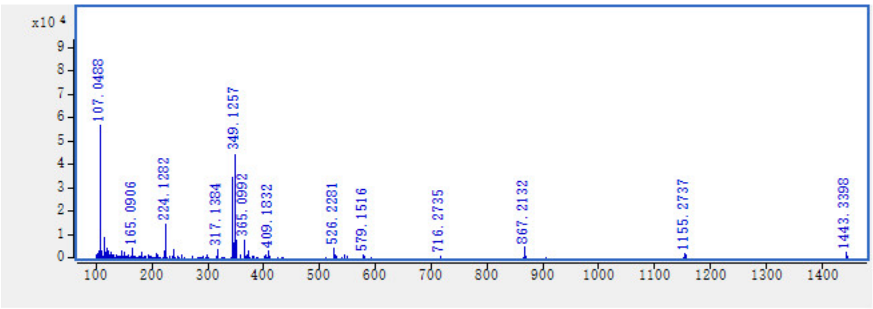

Figure S10 MS spectrum of peak 6

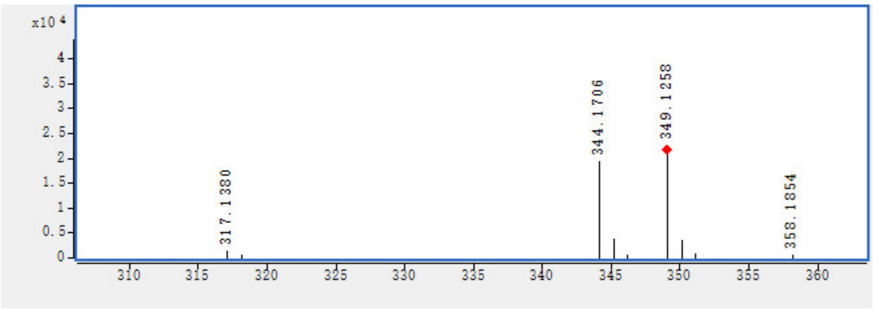

Figure S11 MS/MS spectrum of peak 6

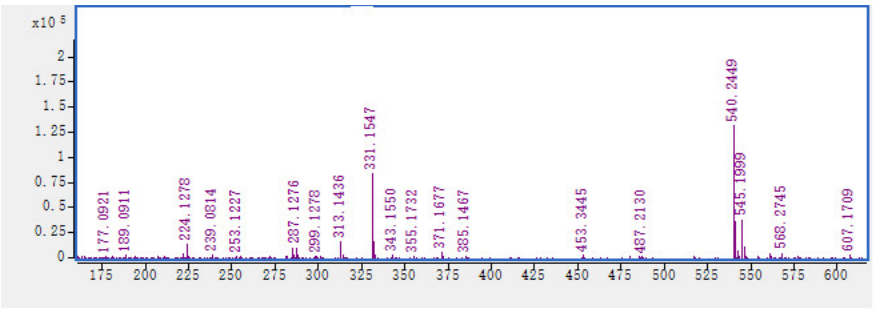

Figure S12 MS spectrum of peak 7

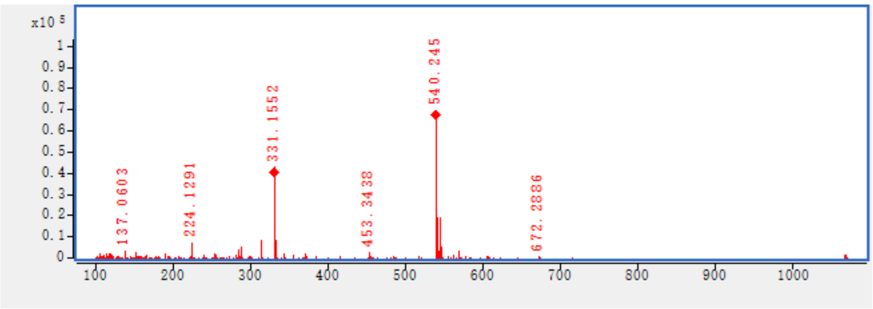

Figure S13 MS/MS spectrum of peak 7

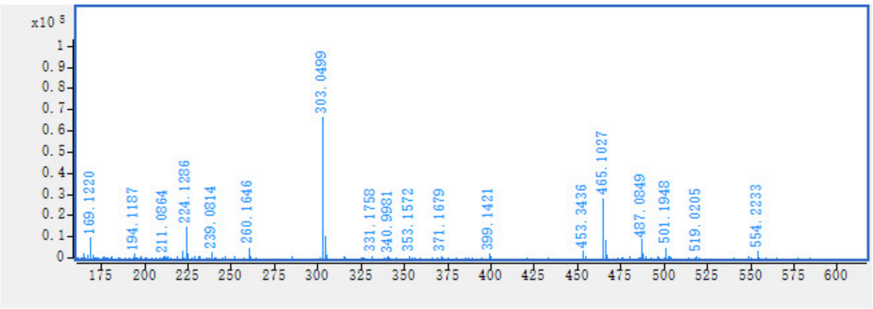

Figure S14 MS spectrum of peak 8

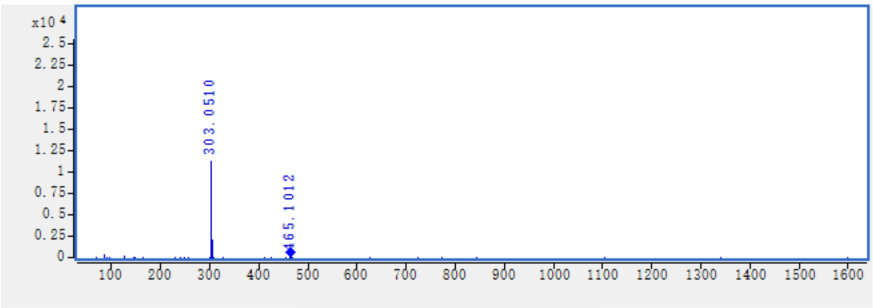

Figure S15 MS/MS spectrum of peak 8

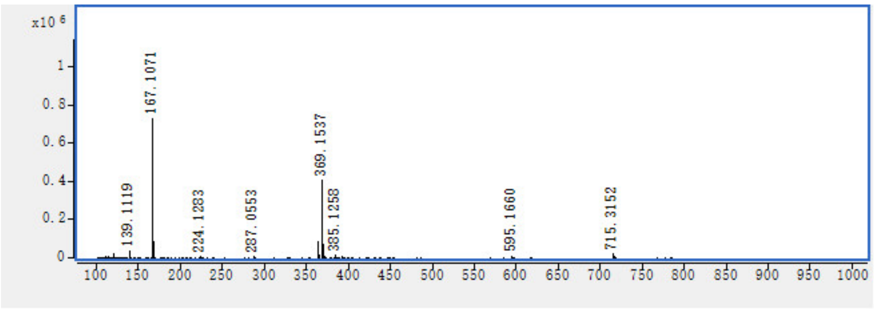

Figure S16 MS spectrum of peak 9

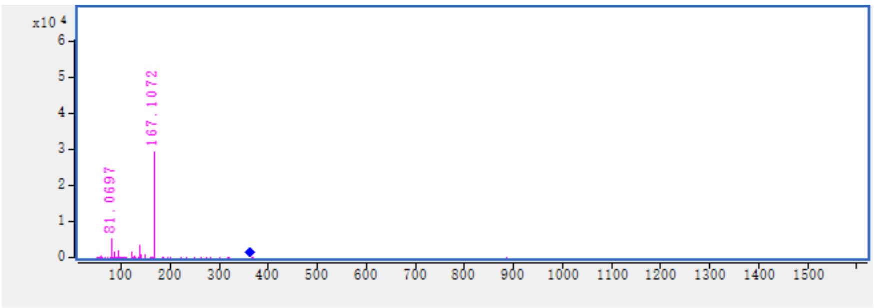

Figure S17 MS/MS spectrum of peak 9

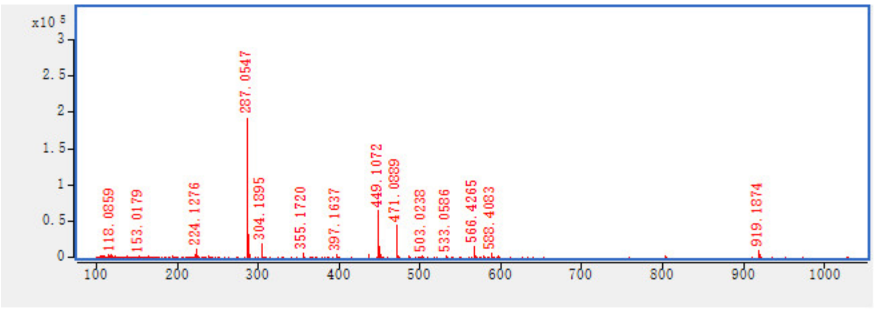

Figure S18 MS spectrum of peak 10

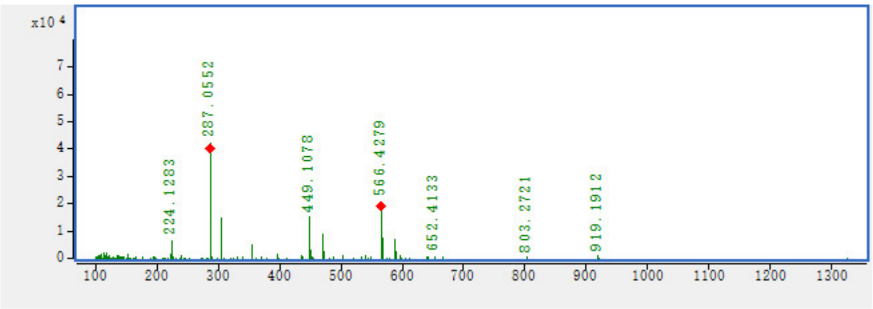

Figure S19 MS/MS spectrum of peak 10

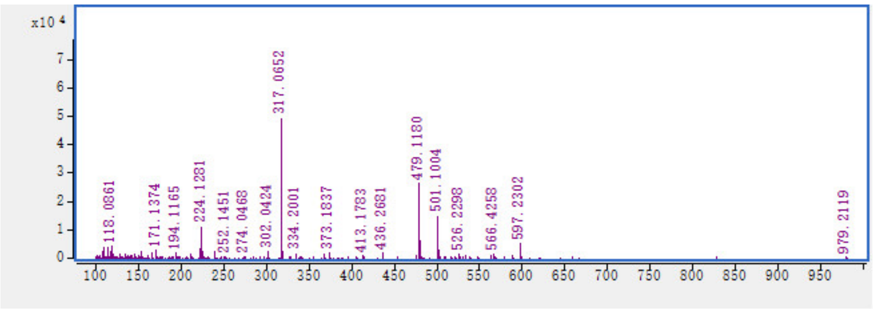

Figure S20 MS spectrum of peak 11

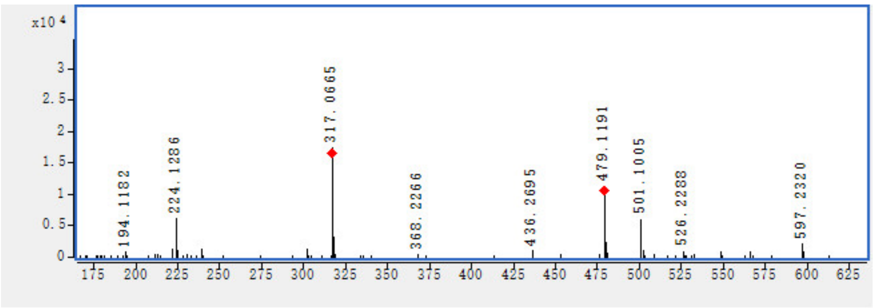

Figure S21 MS/MS spectrum of peak 11

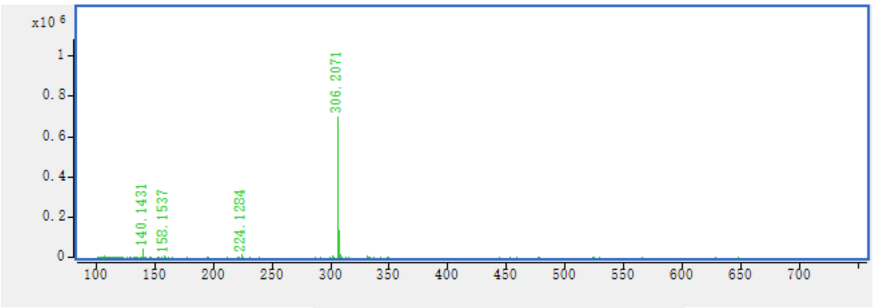

Figure S22 MS spectrum of peak 12

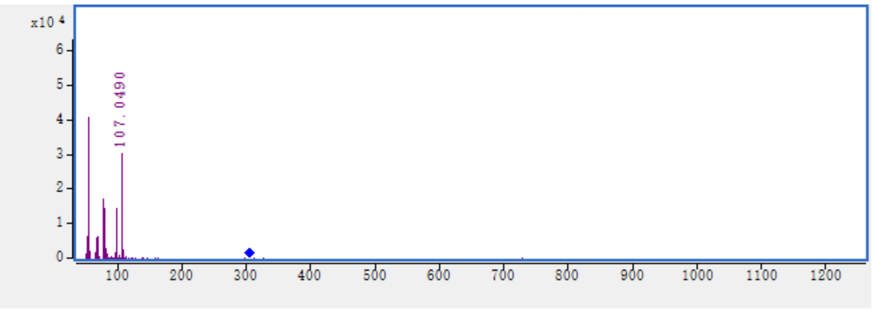

Figure S23 MS/MS spectrum of peak 12

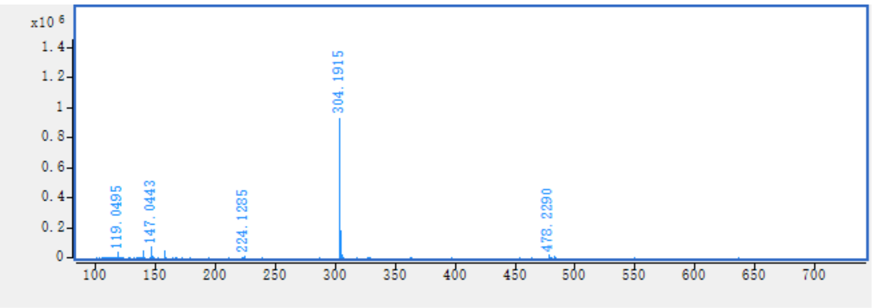

Figure S24 MS spectrum of peak 13

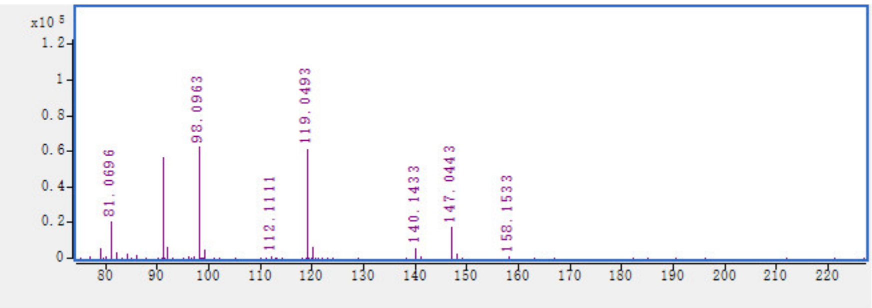

Figure S25 MS/MS spectrum of peak 13

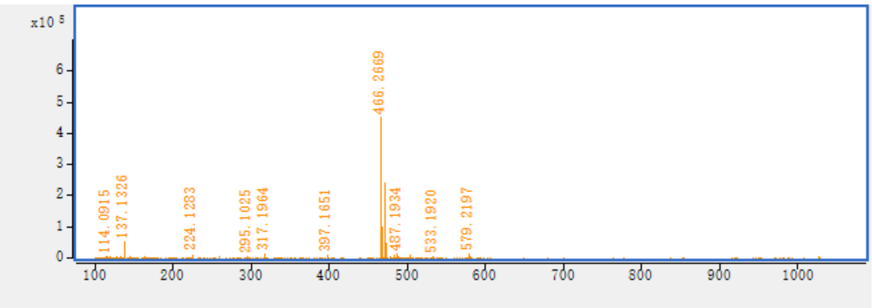

Figure S26 MS spectrum of peak 14

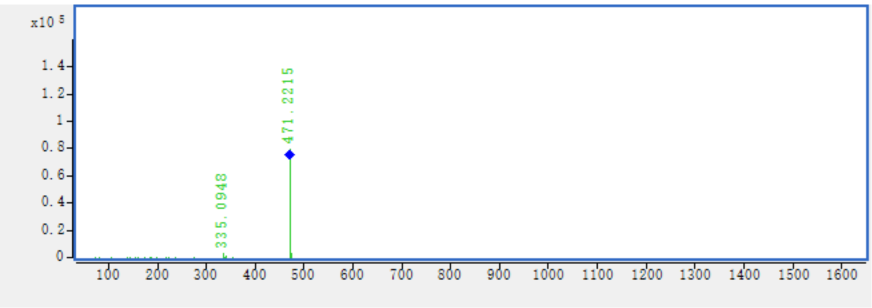

Figure S27 MS/MS spectrum of peak 14

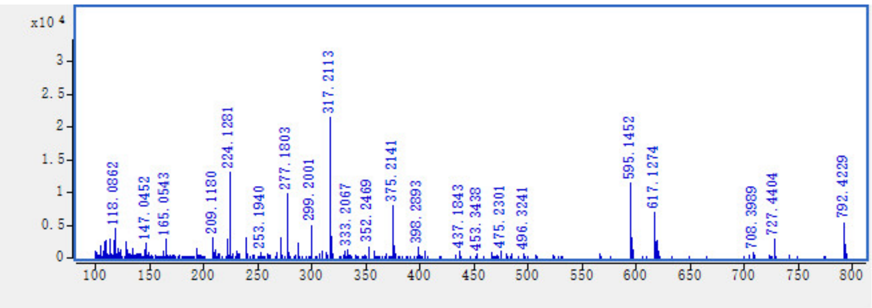

Figure S28 MS spectrum of peak 15

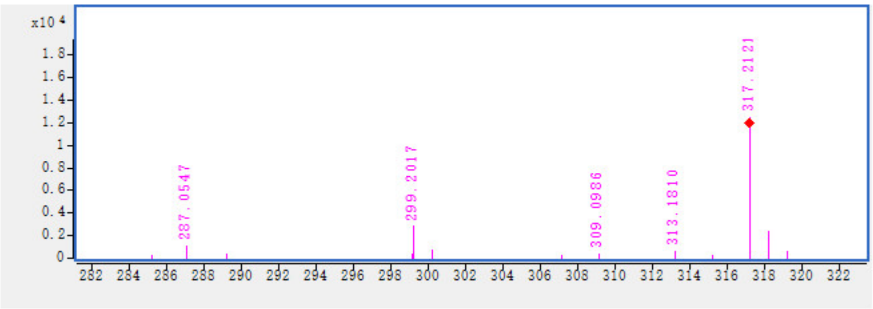

Figure S29 MS/MS spectrum of peak 15

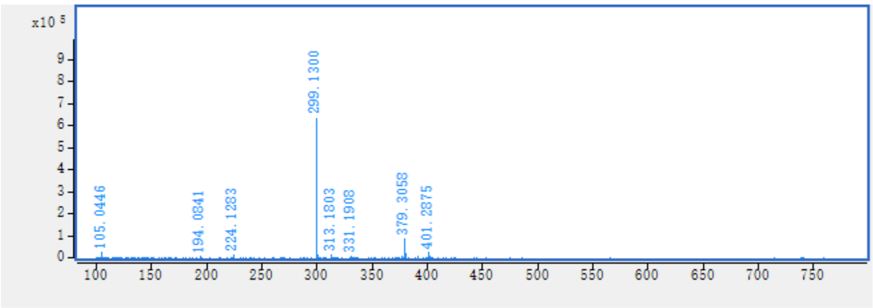

Figure S30 MS spectrum of peak 16

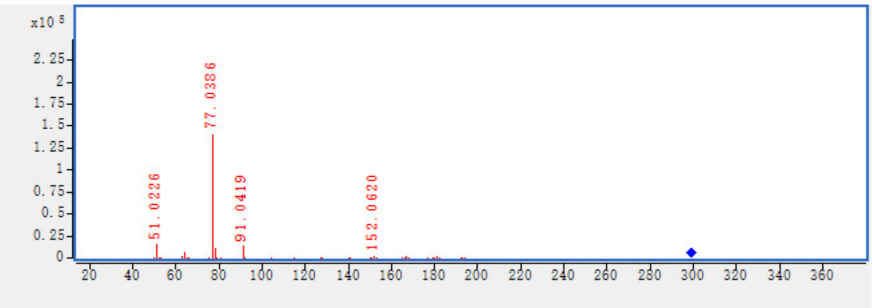

Figure S31 MS/MS spectrum of peak 16

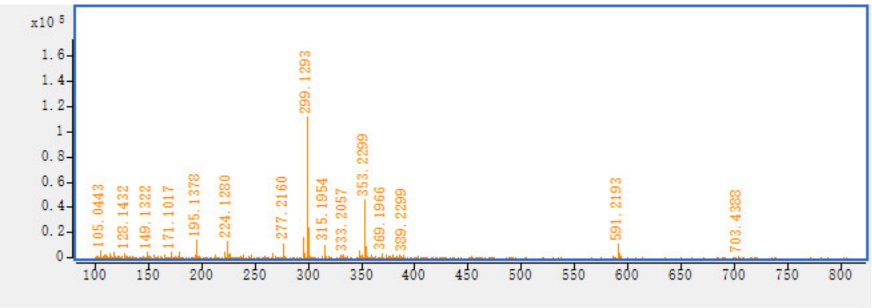

Figure S32 MS spectrum of peak 17

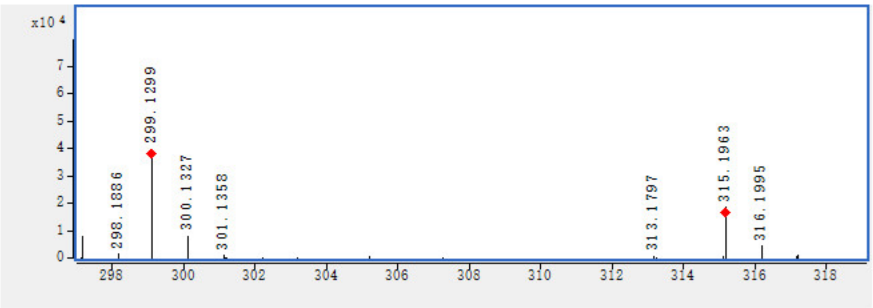

Figure S33 MS/MS spectrum of peak 17

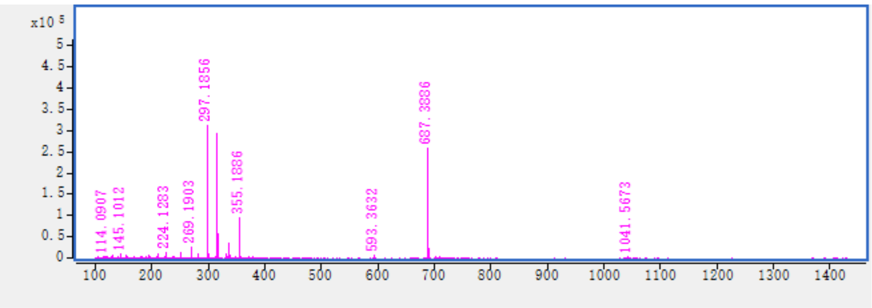

Figure S34 MS spectrum of peak 18

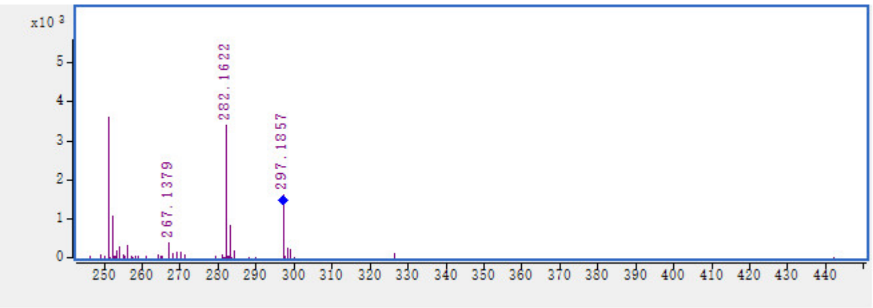

Figure S35 MS/MS spectrum of peak 18

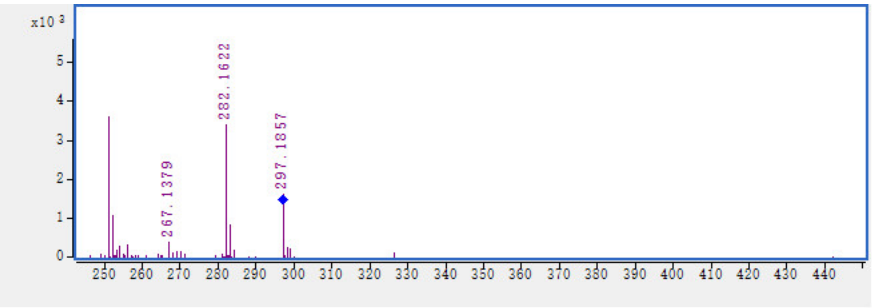

Figure S36 MS spectrum of peak 19

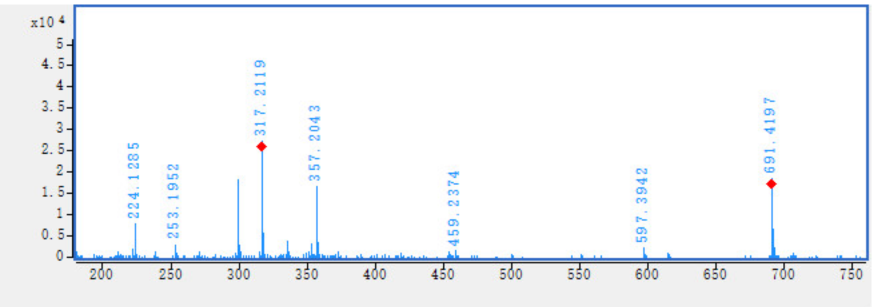

Figure S37 MS/MS spectrum of peak 19

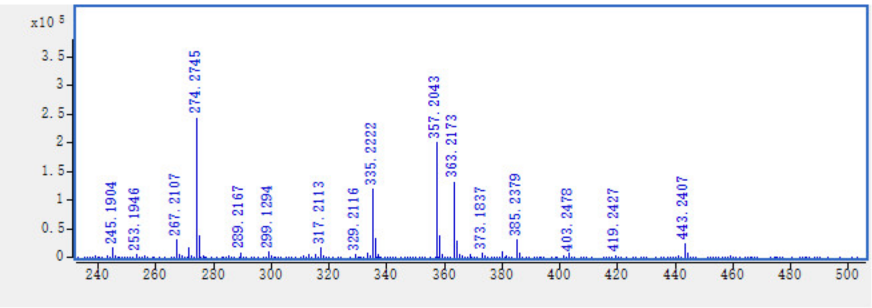

Figure S38 MS spectrum of peak 20

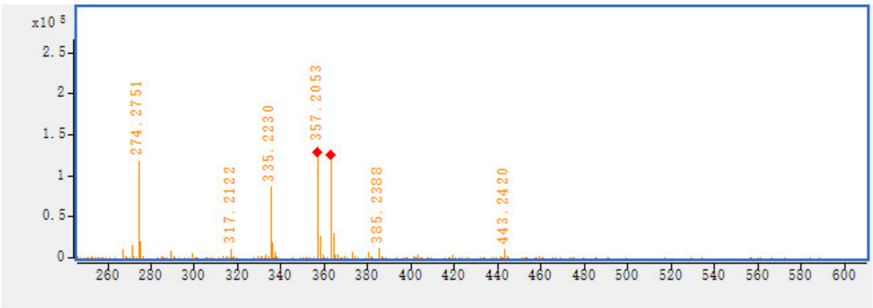

Figure S39 MS/MS spectrum of peak 20

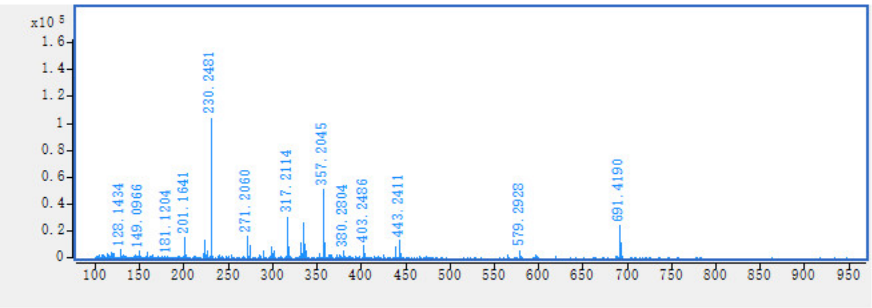

Figure S40 MS spectrum of peak 21

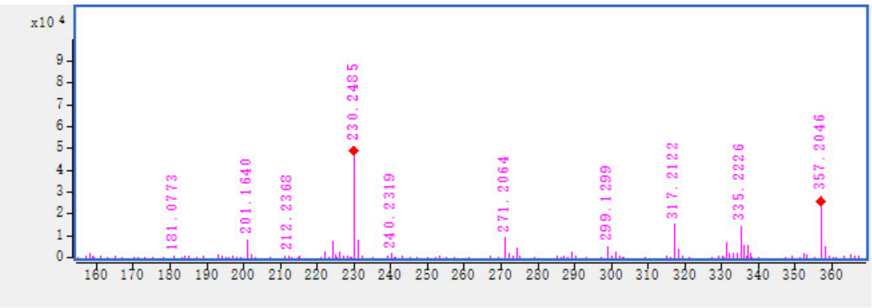

Figure S41 MS/MS spectrum of peak 21

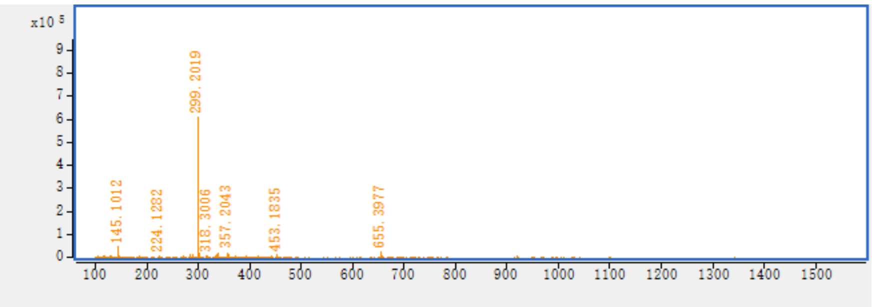

Figure S42 MS spectrum of peak 22

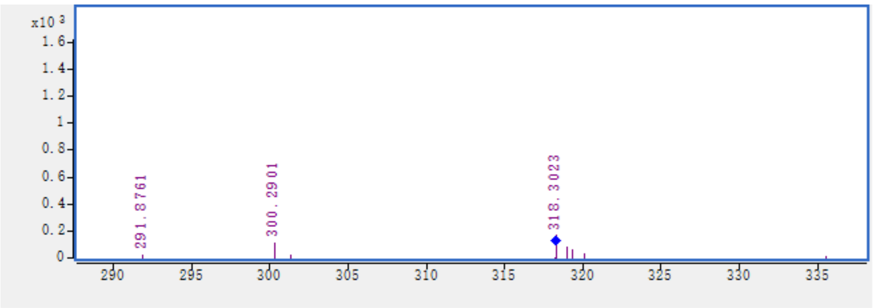

Figure S43 MS/MS spectrum of peak 22

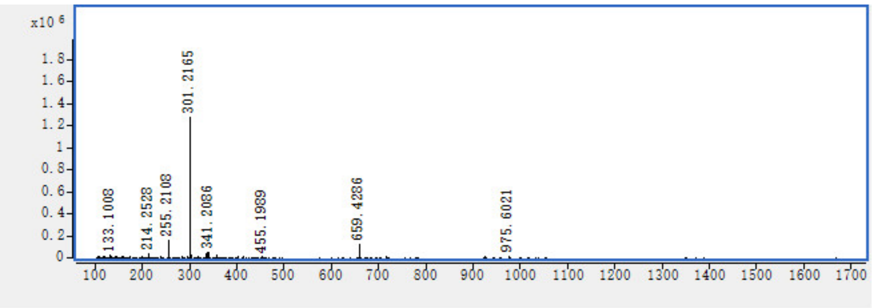

Figure S44 MS spectrum of peak 23

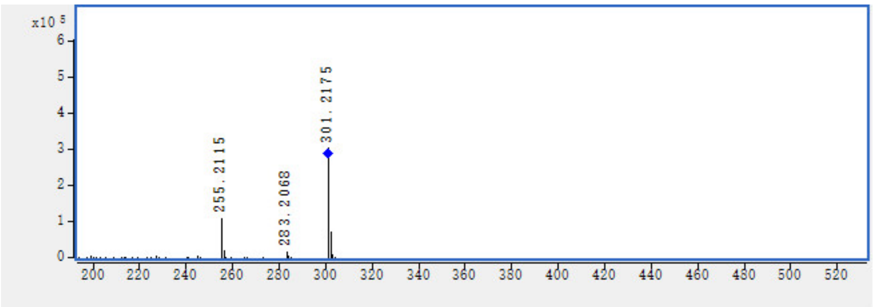

Figure S45 MS/MS spectrum of peak 23

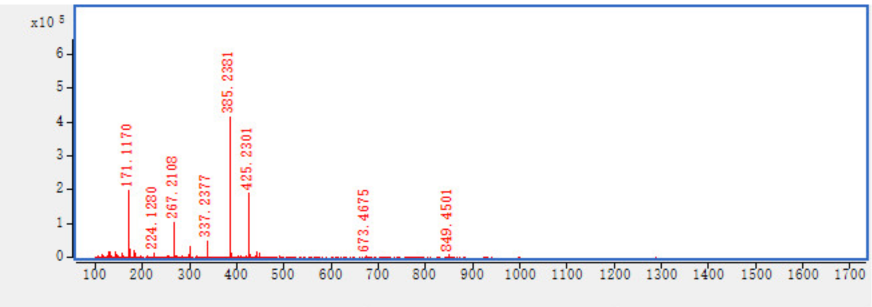

Figure S46 MS spectrum of peak 24

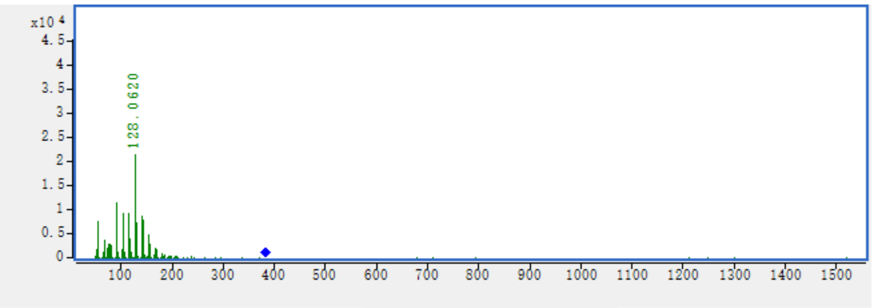

Figure S47 MS/MS spectrum of peak 24

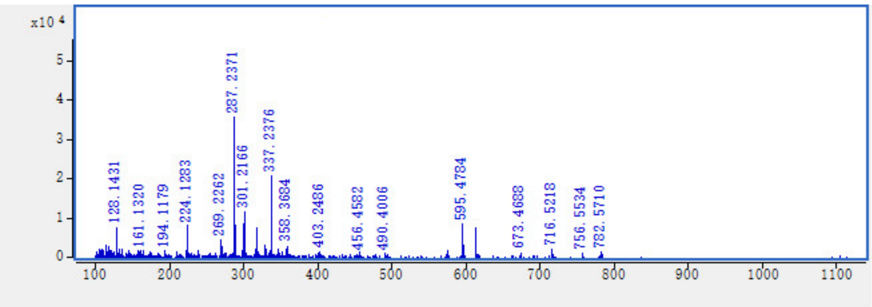

Figure S48 MS spectrum of peak 25

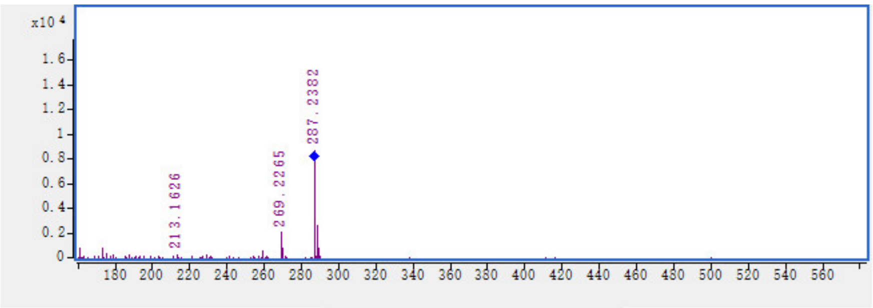

Figure S49 MS/MS spectrum of peak 25

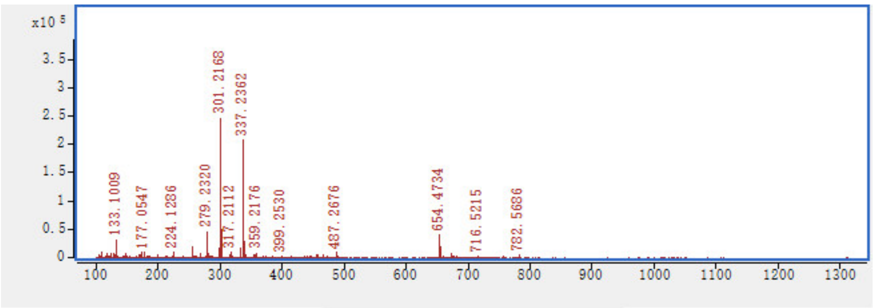

Figure S50 MS spectrum of peak 26

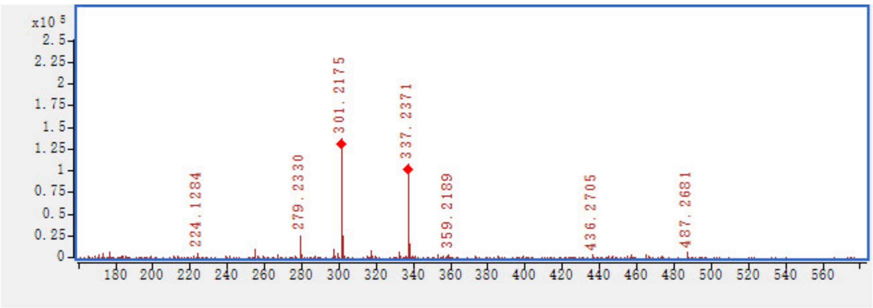

Figure S51 MS/MS spectrum of peak 26

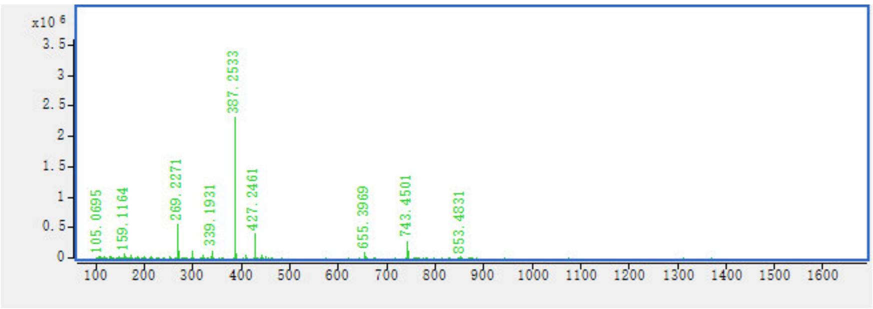

Figure S52 MS spectrum of peak 27

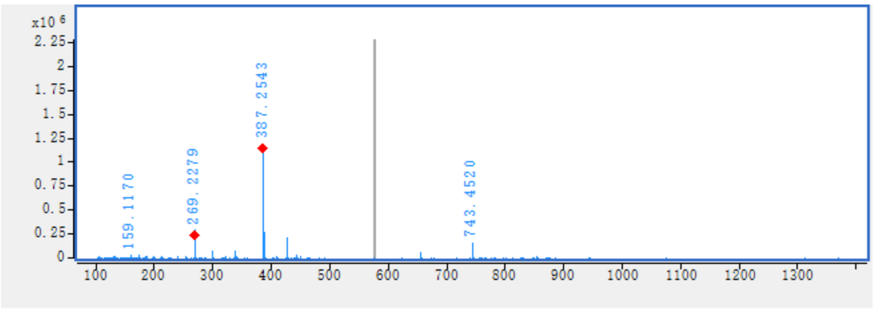

Figure S53 MS/MS spectrum of peak 27

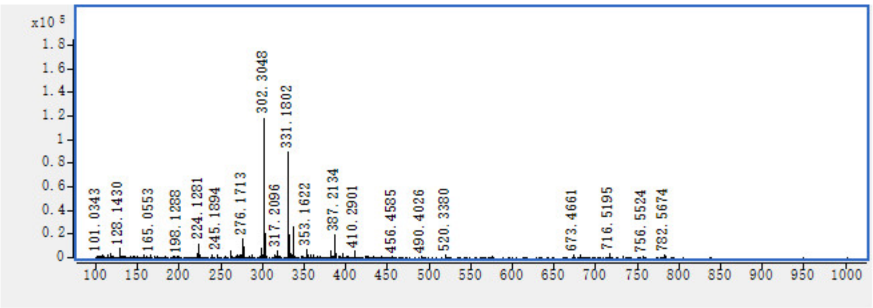

Figure S54 MS spectrum of peak 28

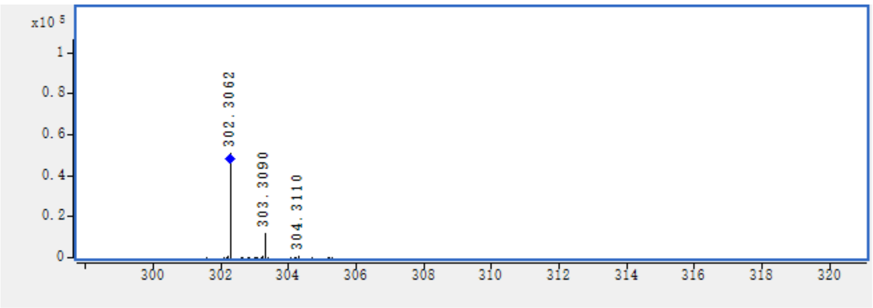

Figure S55 MS/MS spectrum of peak 28

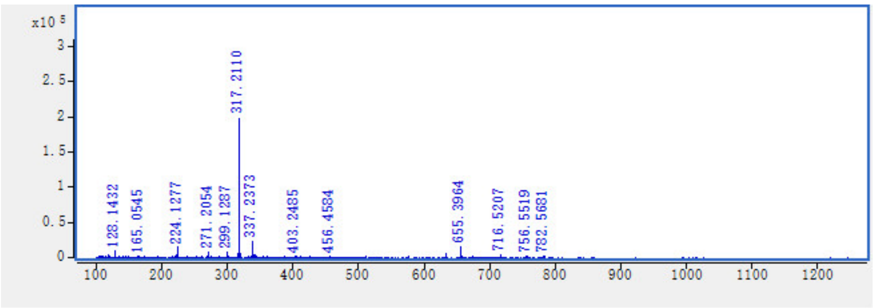

Figure S56 MS spectrum of peak 29

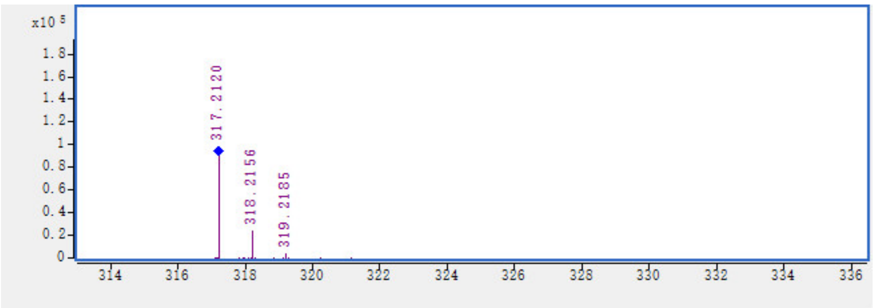

Figure S57 MS/MS spectrum of peak 29

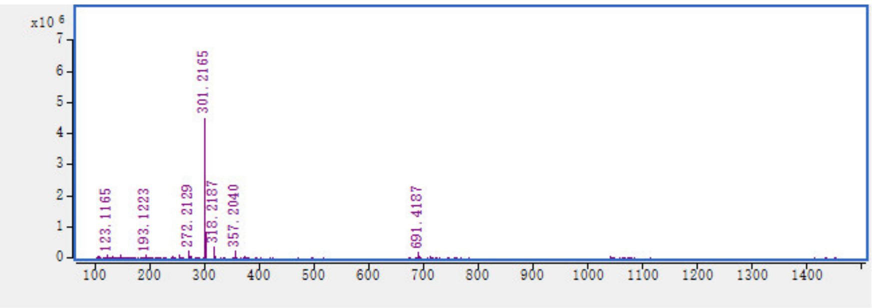

Figure S58 MS spectrum of peak 30

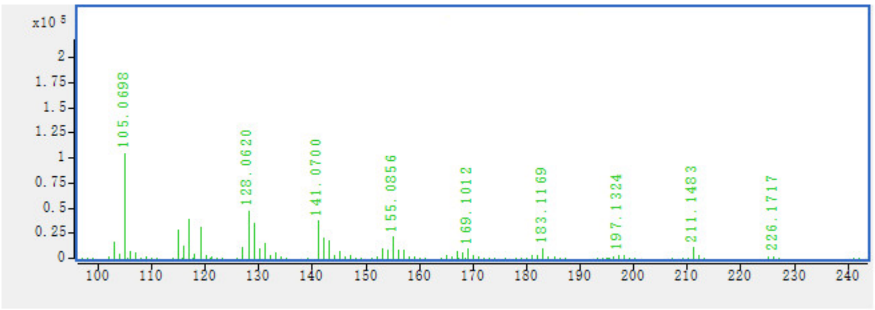

Figure S59 MS/MS spectrum of peak 30

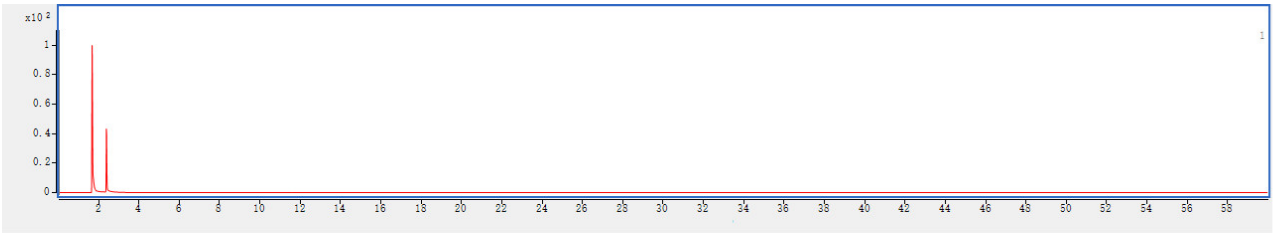

Figure S60 UPLC–MS results captured in positive-ion mode for adenine

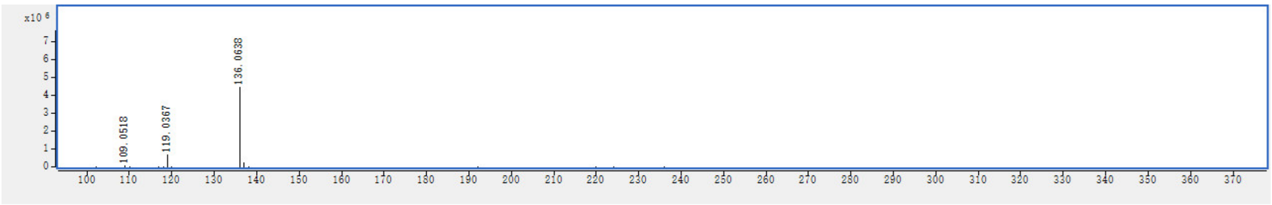

Figure S61 MS spectrum of adenine

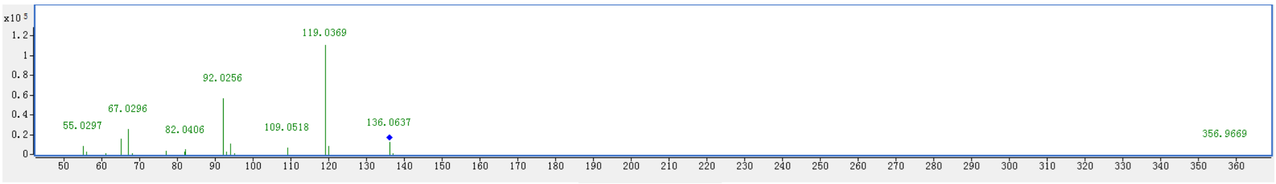

Figure S62 MS/MS spectrum of adenine

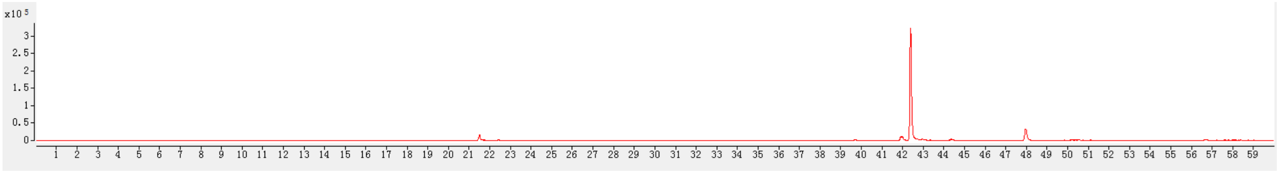

Figure S63 UPLC–MS results captured in positive-ion mode for phytosphingosine

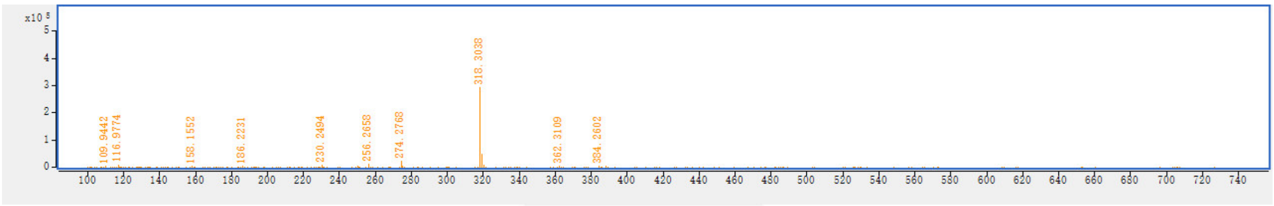

Figure S64 MS spectrum of phytosphingosine

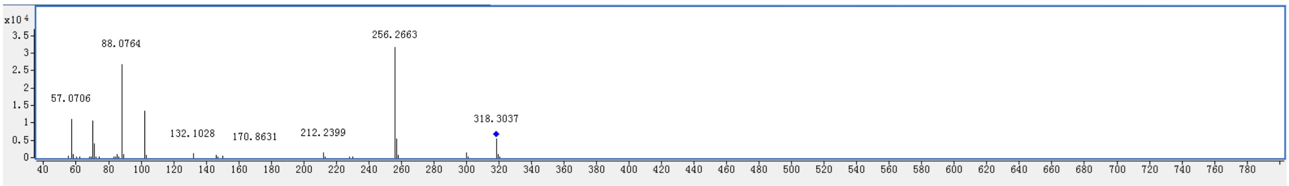

Figure S65 MS/MS spectrum of phytosphingosine

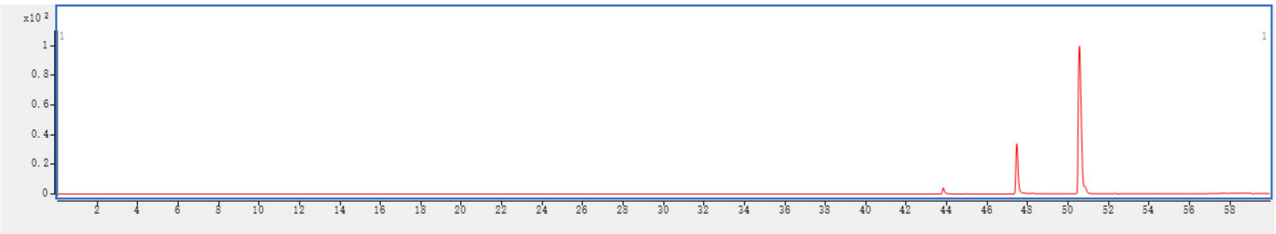

Figure S66 UPLC–MS results captured in positive-ion mode for sphinganine

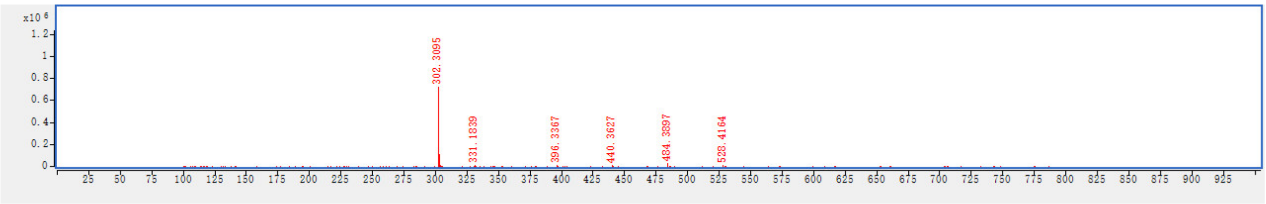

Figure S67 MS spectrum of sphinganine

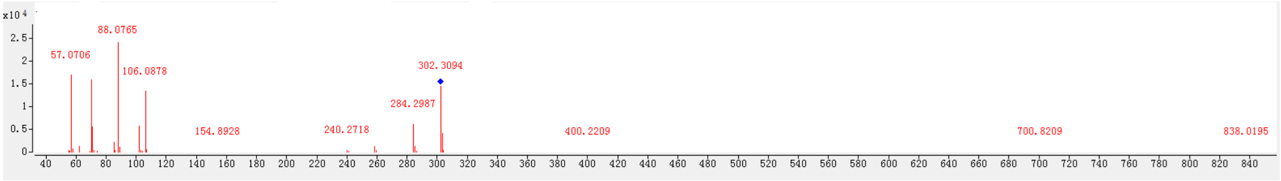

Figure S68 MS/MS spectrum of sphinganine

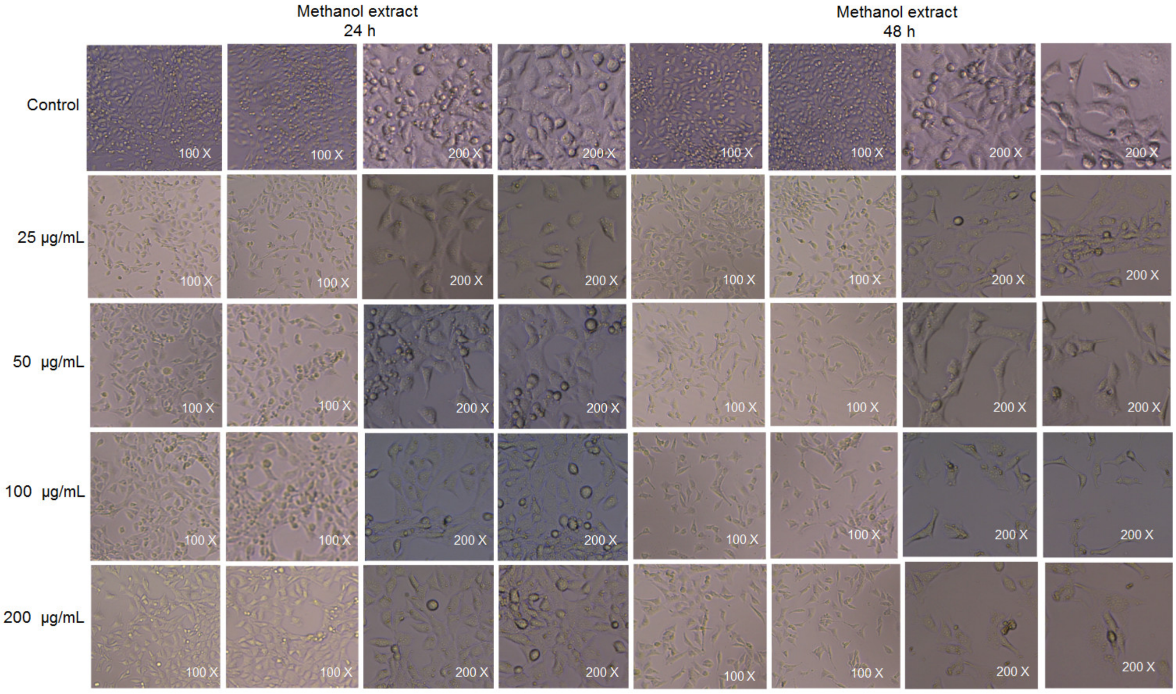

Figure S69 Morphology of TM3 mouse cells treated with methanol extract of insect gall of *Picea koraiensis* for 24 or 48 h

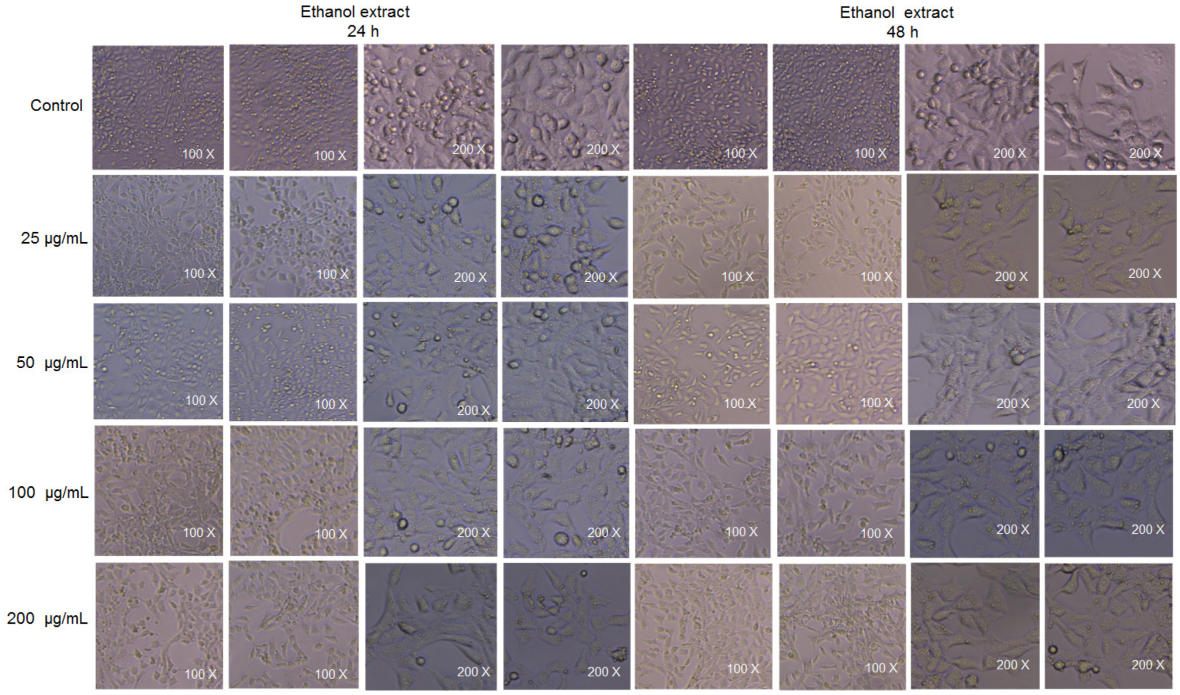

Figure S70 Morphology of TM_3 mouse cells treated with ethanol extract of insect gall of *Picea koraiensis* for 24 or 48 h.
